# Emerging Multifunctional Biomaterials for Addressing Drug Resistance in Cancer

**DOI:** 10.3390/biology14050497

**Published:** 2025-05-02

**Authors:** Mohamed El-Tanani, Syed Arman Rabbani, Rasha Babiker, Yahia El-Tanani, Shakta Mani Satyam, Thantrira Porntaveetus

**Affiliations:** 1 RAK College of Pharmacy, RAK Medical and Health Sciences University, Ras Al Khaimah 11172, United Arab Emirates; 2 RAK College of Medical Sciences, RAK Medical and Health Sciences University, Ras Al Khaimah 11172, United Arab Emirates; 3 Royal Cornwall Hospital Trust, NHS, Truro TR1 3LJ, UK; 4 Center of Excellence in Precision Medicine and Digital Health, Department of Physiology, Faculty of Dentistry, Chulalongkorn University, Bangkok 10330, Thailand; thantrira.p@chula.ac.th

**Keywords:** biomaterials, cancer, drug resistance, electrical modulation, catalytic nanomaterials, tumor microenvironment, reactive oxygen species, precision oncology

## Abstract

Drug resistance remains a major barrier to effective cancer treatment, contributing to poor patient outcomes. Multifunctional biomaterials integrating electrical and catalytic properties offer a transformative strategy to target diverse resistance mechanisms. This review explores their ability to modulate cellular processes, remodel the tumor microenvironment (TME), and enhance drug delivery. Electrically active biomaterials enhance drug uptake and apoptotic sensitivity by altering membrane potentials, ion channels, and intracellular signaling, synergizing with chemotherapy. Catalytic biomaterials generate reactive oxygen species (ROS), activate prodrugs, reprogram hypoxic and acidic TME, and degrade the extracellular matrix (ECM) to improve drug penetration. Hybrid nanomaterials (e.g., conductive hydrogels, electrocatalytic nanoparticles), synergize electrical and catalytic properties for localized, stimuli-responsive therapy and targeted drug release, minimizing systemic toxicity. Despite challenges in biocompatibility and scalability, future integration with immunotherapy, personalized medicine, and intelligent self-adaptive systems capable of real-time tumor response promises to accelerate clinical translation. The development of these adaptive biomaterials, alongside advancements in nanotechnology and AI-driven platforms, represents the next frontier in precision oncology. This review highlights the potential of multifunctional biomaterials to revolutionize cancer therapy by addressing multidrug resistance at cellular, genetic, and microenvironmental levels, offering a roadmap to improve therapeutic outcomes and reshape oncology practice.

## Simple Summary

Cancer treatments often fail because cancer cells become resistant to drugs, leading to poor survival rates. To address this, scientists are developing advanced materials called biomaterials that combine unique properties like electrical activity and catalytic (reaction-boosting) abilities. These materials help overcome resistance by making cancer cells more responsive to treatment. For example, some biomaterials use tiny electrical signals to open cancer cell membranes, allowing drugs to enter more easily. Others produce molecules that stress cancer cells, making them weaker and more likely to die. These materials can also break down the dense environment around tumors, helping drugs reach deeper into the cancer. By combining these strategies, researchers create “smart” materials that release drugs only where needed, reducing side effects. However, challenges remain in making these materials safe for the body and scalable for widespread use. Future work aims to pair them with immunotherapy (treatments that use the body’s immune system) and personalized medicine to tailor therapies to individual patients. If successful, these biomaterials could transform cancer care by making treatments more effective, less toxic, and adaptable to each person’s unique cancer. This progress offers hope for improving survival and quality of life for millions affected by drug-resistant cancers worldwide.

## 1. Introduction

Cancer is one of the most common causes of death worldwide, and the World Health Organization (WHO) estimates that it kills about 10 million people every year [1]. Cancer remains one of the deadliest diseases, even with the current techniques of diagnosis and treatment. The efficacy of chemotherapy and other treatments, such as targeted therapies and immunotherapies [2], is limited by intrinsic or acquired drug resistance [3]. Hence, suboptimal patient results, caused by treatment failure and disease progression, are of major interest in terms of the processes that underlie developing drug resistance and new methods of combating it to enhance the efficacy of cancer treatments.

Conventional chemotherapies and some targeted therapies often suffer from limited efficacy due to the development of drug resistance. Immunotherapies, although promising and generally unaffected by classical drug resistance mechanisms, may still face challenges related to immune evasion and tumor heterogeneity [4]. However, several treatment strategies, particularly nanoparticle-based delivery systems, demonstrate high efficacy in tumors with low-grade malignancy, validating the importance of tumor type and its role in therapeutic responsiveness [5].

To address the challenge of drug resistance in cancer treatment, efforts are underway to develop inhibitors that work together in targeting pathways and reshaping the tumor microenvironment (TME). While these innovative approaches have shown potential in both lab and clinical trials, it is worth noting that they may lead to side effects affecting biological targets and could eventually result in secondary resistance development [6]. For example, EGFR inhibitors are also utilized in treating non-small cell lung cancer (NSCLC), but there is a risk of developing resistance mutations, like T790M, which may necessitate subsequent rounds of treatment using new drugs [7]. In cases where patients are prescribed a combination of therapeutic interventions, the potential emergence of adverse effects may compromise adherence to the treatment regimen, as delineated by their physician or healthcare professional. That is why it is important to come up with ways that involve different fields of expertise to tackle drug resistance in a more effective and safe manner.

One promising scientist-recommended approach uses biomaterials, which serve as a solution to this problem while increasing the effectiveness of existing treatments. New types of biomaterials are emerging that have characteristics such as conductivity [8] and catalytic activity [9] combined with biocompatibility in one material known as a multifunctional biomaterial. A diverse range of functions within a single system has been observed to be crucial in the medical field for cancer treatment. Promising attributes have been discovered, and they help in creating more advanced treatment methods by incorporating various functionalities. The membrane potential is altered by conductive materials such as polymers [10] and nanoparticles [11], followed by alteration in cancer cell behavior [12], enhancing drug absorption and thus increasing the efficacy of chemotherapy. They control the bioelectric signals and use catalytic nanomaterials to produce reactive oxygen species (ROS) to make cancer cells more sensitive [13]. These materials also alter the TME to overcome resistance and switch on prodrugs, with reduced side effects [14].

The fusion of catalytic characteristics into biomaterials can address the complex issue of drug resistance effectively by leveraging these properties to target multiple levels simultaneously at the cellular level, genetic level, and microenvironment level, which play key roles in the resistance development process [15]. For example, nanostructures capable of generating ROS and administering electric stimuli can combat cell signaling pathways and the tumor microenvironment concurrently, supporting cancer treatment outcomes [16]. This article explores the utilization of biomaterials to address drug resistance in cancer therapy by examining their mechanisms and potential impact on the future of cancer treatment and the well-being of patients battling cancer. In the future, ongoing interdisciplinary research in this field, encompassing material science and nanotechnology alongside oncology, could potentially offer solutions to this issue in cancer therapy.

## 2. Drug Resistance Mechanisms in Cancer

### 2.1. Tumor Heterogeneity

**Figure 1:**
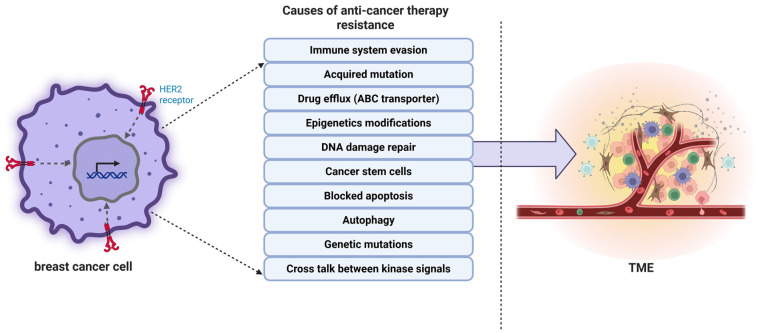
Illustrates the multiple mechanisms by which HER2-positive breast cancer cells develop resistance to anti-cancer therapies. These include immune system evasion, acquired mutations, drug efflux via ATP-binding cassette (ABC) transporters, epigenetic modifications, DNA damage repair, cancer stem cell survival, inhibition of apoptosis, autophagy, and genetic mutations. Furthermore, crosstalk between kinase signaling pathways contributes to treatment failure. These resistance mechanisms not only sustain tumor cell survival but also influence the tumor microenvironment (TME), facilitating angiogenesis, immune suppression, and tumor progression. Created in BioRender. Babiker, R. (2025) https://BioRender.com/9inm7ec, accessed on 27 April 2025.

As previously reported, 90% of failures have been shown in chemotherapy during the metastasis of cancer, related to drug resistance. Many patients’ tumor cells develop drug resistance during chemotherapy due to the administration of a certain drug [17]. Resisting cancer drugs involves a process, with factors at play that explain how cancer cells become unresponsive to treatment methods over time (Figure 1). There are two types of intrinsic resistance: resistance (in which tumors are naturally resistant to treatment because of epigenetic factors like miRNA alteration, transcriptomic or proteomic heterogeneity, etc.) [18,19] and acquired resistance (in which cancer cells develop resistance during treatment as they adapt to the therapy due to pH, hypoxia, etc.) [20]. These mechanisms operate at the molecular level. They often work together to present a significant hurdle in the field of clinical oncology. Key elements that lead to resistance to drugs include efflux pumps, changes in drug targets, signaling pathways related to cell death, processes like DNA repair, and epigenetic and metabolic changes [21]. All of these work together to reduce the effectiveness of treatments along with aberrant nuclear export mechanisms have been increasingly recognized as contributors to chemotherapy resistance in cancers, interfering with nuclear-cytoplasmic transport of tumor suppressor proteins [22].

There are various signaling pathways involved in tumor progression and drug resistance, including the Wnt pathway. Alterations in Wnt-β-catenin signaling pathways, modulated by osteopontin expression, contribute to enhanced invasiveness and therapy resistance in breast cancer cells [23].

Efflux pumps are widely acknowledged as key players in the development of drug resistance in the field. These pumps are categorized under a group of membrane proteins called ATP-binding cassette (ABC) transporters, with P-glycoprotein being a member among them. They work to expel chemotherapy agents from cancer cells, lowering their levels within the cells and reducing their effectiveness. Important efflux transporters include multidrug resistance-associated proteins (MRPs) and breast cancer resistance protein (BCRP) [24]. These pumps are frequently seen at elevated levels after extended periods of chemotherapy and are followed by increasing resistance to multiple drugs (MDR). Blocking these pumps is possible with verapamil and cyclosporine, but it is difficult due to a lack of precision in targeting specific pumps [25]. Furthermore, there arises the issue of changes in the targets on which the drugs act. It is common for cancer cells to undergo mutations, which leads to alteration in the expression of proteins and pathways that control their functions. For instance, alterations in the epidermal growth factor receptor (EGFR), such as the T790M mutation, cause resistance to tyrosine kinase inhibitors (TKIs) [26]. Similarly, mutations in the HER2 or BCR-ABL kinase domain, like T315I, result in resistance in breast cancer and chronic myeloid leukemia (CML), respectively [27].

In addition to the mechanisms involving mitochondrial-mediated apoptosis, tumor heterogeneity also plays a significant role in modulating other apoptotic pathways that contribute to drug resistance. One such pathway is the FAS-mediated (extrinsic) apoptotic pathway, which, like its mitochondrial counterpart, can be suppressed in resistant cancer cells. Emerging evidence suggests that the downregulation of Fas ligand (FasL) expression is associated with acquired resistance to chemotherapeutic agents such as cisplatin [28]; cisplatin-resistant human ovarian cancer cells exhibit sustained suppression of FasL expression, thereby impairing apoptosis via the FAS pathway. This resistance mechanism is particularly concerning, as it highlights how cancer cells can escape immune-mediated cell death and resist therapies that rely on extrinsic apoptotic signaling [29].

A key process in treatment resistance involves alterations that allow cancer cells to avoid programmed cell death, known as apoptosis. In resistant cancer cells, apoptosis is often inhibited by increased levels of anti-apoptotic proteins, such as BCL-2 and BCL-XL, which block the mitochondrial pathway of cell death [30]. Conversely, downregulating the levels of pro-apoptotic proteins like BAX and BAK can impair cell death signaling [31]. Additionally, mutations in the TP53 gene, which encodes the p53 tumor suppressor protein, can compromise its ability to trigger apoptosis and respond to DNA damage, leading to resistance against DNA-damaging agents [32].

Epigenetic changes, including DNA methylation and alterations in non-coding RNAs, cause drug resistance by modifying gene expression [33]. Metabolic reprogramming, like the Warburg effect and altered glutamine metabolism, increases cancer cell survival and resistance to therapies, including immunotherapy [34]. Genetic mutations involving oncogenes such as MYC and associated regulatory proteins like RanGTPase contribute to drug resistance and promote cancer progression [35].

**Figure 2:**
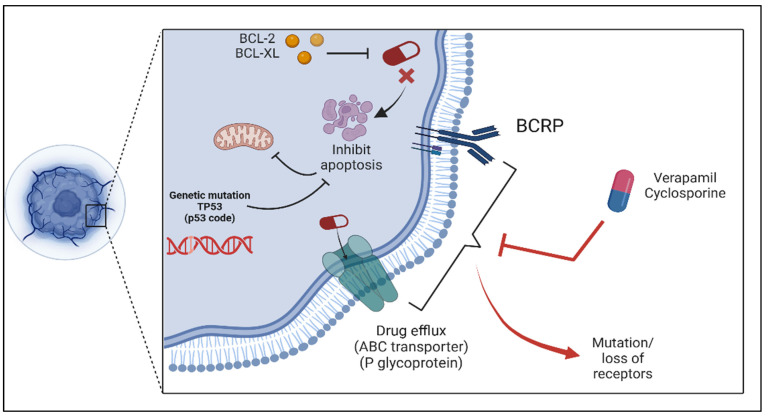
Key mechanisms of drug resistance in cancer cells, including apoptosis inhibition and drug efflux. Genetic mutations, such as TP53 (p53 code) mutations, lead to apoptosis resistance by disrupting mitochondrial pathways. Overexpression of BCL-2 and BCL-XL proteins further inhibits apoptosis, preventing cancer cell death. Additionally, ABC transporters like P-glycoprotein and BCRP (Breast Cancer Resistance Protein) actively pump drugs out of the cell, reducing their efficacy. Verapamil and cyclosporine are shown as inhibitors targeting drug efflux mechanisms. Mutations or loss of drug receptors further contribute to treatment resistance, limiting drug binding and effectiveness. Created in BioRender. Babiker, R. (2025) https://BioRender.com/1ctpwf3, accessed on 27 April 2025.

Understanding these interconnected pathways is important for developing strategies against resistance to cancer, potentially by utilization of biomaterials having electrical and catalytic properties, to combat resistance on multiple fronts. These approaches promise to advance cancer treatment by addressing both genetic and environmental factors, influencing tumor behavior and response to therapy (Figure 2, Table 1).

### 2.2. Role of Tumor Microenvironment (TME) in Drug Resistance

The tumor microenvironment provides a shield, promoting drug resistance through dynamic interactions among supportive cells and growth factors [52]. Hypoxia is a situation in the tumor microenvironment caused by insufficient blood supply and rapid growth of tumor cells; it triggers the HIF-α gene, which regulates the expression of genes promoting resistance to medications [53]. These genes produce pumps that expel chemotherapy drugs from cells and proteins that prevent cell death caused by treatment. Moreover, under low-oxygen conditions, cells utilize anabolic glycolysis more, reducing the effectiveness of oxygen-dependent treatments, such as radiation and certain chemotherapy drugs [54]. This complicates the task of treating regions within tumors, ultimately leading to the development of aggressive tumor characteristics as a result of resistance induced by hypoxia.

Tumor acidity, driven by lactate production, impairs pH-dependent drugs, fostering cancer cell survival and immune evasion. Furthermore, the extracellular matrix (ECM) acts as a physical barrier, restricting drug penetration while activating survival pathways through interactions with cancer cells [55].

The extracellular matrix (ECM) within the tumor microenvironment (TME) acts as a barrier that impedes the delivery of drugs due to its composition, mainly consisting of collagen and other substances like glycoproteins and proteoglycans. It restricts the entry of treatment agents into the core of the tumor by interacting with integrins and receptors on cancer cells to trigger signaling pathways associated with enhanced cell survival, growth, and resistance to therapy [56]. Furthermore, the extracellular matrix (ECM) serves as a framework for both tumor and stromal cells. When it breaks down, it releases growth factors that support cell survival and the formation of blood vessels, which can counteract the effectiveness of the treatment.

New versatile biomaterial technologies have emerged as solutions for reshaping the tumor microenvironment (TME), despite the inherent difficulties it presents. One notable example is the use of oxygen-generating nanomaterials to counteract hypoxia, thereby improving treatment efficacy, while pH-regulating materials enhance drug uptake [57]. ECM-disrupting biomaterials, such as enzymatic nanoparticles, facilitate drug delivery and hinder tumor–stroma interactions.

**Figure 3:**
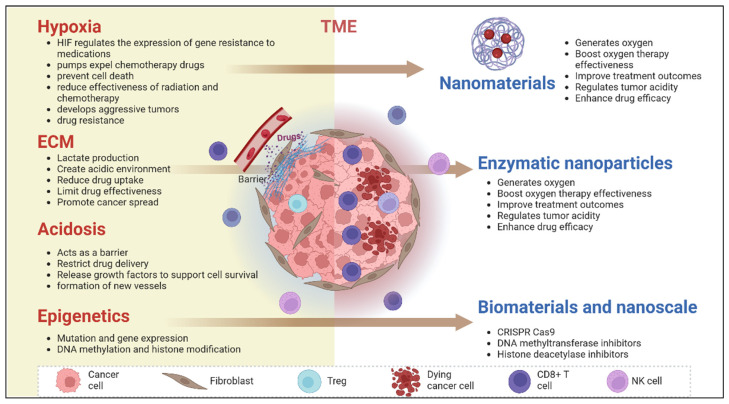
The tumor microenvironment (TME) and the role of biomaterials in overcoming treatment resistance. The TME contributes to drug resistance through the following aspects. Hypoxia: The low-oxygen conditions in tumors activate hypoxia-inducible factors (HIFs), which upregulate genes involved in drug resistance and promote efflux pumps that expel chemotherapy agents. This suppresses cell death, reduces radiotherapy and chemotherapy efficacy, fosters aggressive tumor behavior, and facilitates resistance. Extracellular matrix (ECM): The tumor ECM contributes to lactate production and acidic pH, creating a hostile environment that limits drug uptake and effectiveness while facilitating cancer cell invasion and metastasis. Acidosis: The acidic conditions of the TME act as physical and biochemical barriers, restricting drug penetration. It also promotes angiogenesis and supports tumor survival through growth factor release. Epigenetic modifications: Aberrant gene expression caused by DNA methylation and histone modifications leads to tumor progression and therapeutic resistance. Nanomaterials, enzymatic nanoparticles, and biomaterials at the nanoscale aim to enhance treatment efficacy by generating oxygen, regulating tumor acidity, and incorporating CRISPR-Cas9 systems, DNA methyltransferase inhibitors, and histone deacetylase inhibitors into nano-platforms, allowing for targeted epigenetic reprogramming, overcoming resistance and improving treatment precision. Created in BioRender. Babiker, R. (2025) https://BioRender.com/mo1lvv0, accessed on 27 April 2025.

Resistance mechanisms also stem from genetic and epigenetic changes, including apoptosis dysregulation and drug efflux. Biomaterials incorporating CRISPR-Cas9 or epigenetic modulators can potentially reverse resistance, offering personalized therapeutic strategies to enhance cancer treatment outcomes [58]. These biomaterials could also alter cancer treatment, potentially unlocking personalized therapeutic methods in the future (Figure 3).

## 3. Multifunctional Biomaterials

Multifunctional biomaterials are designed to incorporate several properties, such as conductivity and catalytic activity, and are expected to play a synergistic role in biological functions [59]. At present, these materials are used in biomedical diagnosis, treatment, and tissue engineering. The ability of these materials to address complex medical challenges, such as precise cancer treatment, tissue regeneration, and wound healing, is attributed to their combined electrical and catalytic properties [60].

Another major advantage of these materials is their electrical conductivity, which is useful in applications like neural interfaces, cardiac patches, and wound healing, in which electrical stimulation is known to affect cell functions such as proliferation, differentiation, and migration. These materials’ catalytic activity enables biochemical reactions, including the generation of reactive oxygen species for cancer treatment or the degradation of toxic molecules in their vicinity [61]. Each of these characteristics enhances the effectiveness of multifunctional biomaterials in therapy management, providing better control.

Nanomaterials are the most representative examples of multifunctional biomaterials. Graphene and carbon nanotube-based nanostructures are conductive and have been applied in drug delivery and biosensing systems. Owing to their surface area and functionalization, they have been used in cancer treatment and tissue regeneration [62]. Advanced materials, like MXenes—metallic 2D materials with high hydrophilicity, are being investigated for their application in enhanced wound healing devices [63]. These are particularly well suited for electrical interfacing and catalytic applications and thus are of interest for smart biomedical platforms. Also, nanoencapsulation strategies, such as sophorolipids in PEGylated PLGA, have shown improved therapeutic targeting and bioavailability in colon carcinoma models [64].

Conductive polymers fall into another group that includes substances like polyaniline and polypyrrole, such as PEDOT:PSS, which possess conductivity and can be tailored to incorporate sites. These materials’ flexibility and durability in processing make them ideal for crafting frameworks and layers for biomedical instruments. They stimulate cells, enhancing cellular activity and thereby promoting better tissue regeneration and healing [65].

Metal–organic frameworks (MOFs) represent another group of biomaterials with diverse functional applications. MOFs are structured materials in which metal ions are connected to ligands, forming a framework. These materials can be customized to include active catalytic sites and a conductive network, expanding their applications. The application of nMOFs in cancer treatment has proven successful due to their ability to function as nano-vehicles for precise drug delivery [66]. They are highly effective in enhancing treatment outcomes due to their ability to release therapeutic agents in response to the tumor-specific stimuli environment.

**Figure 4:**
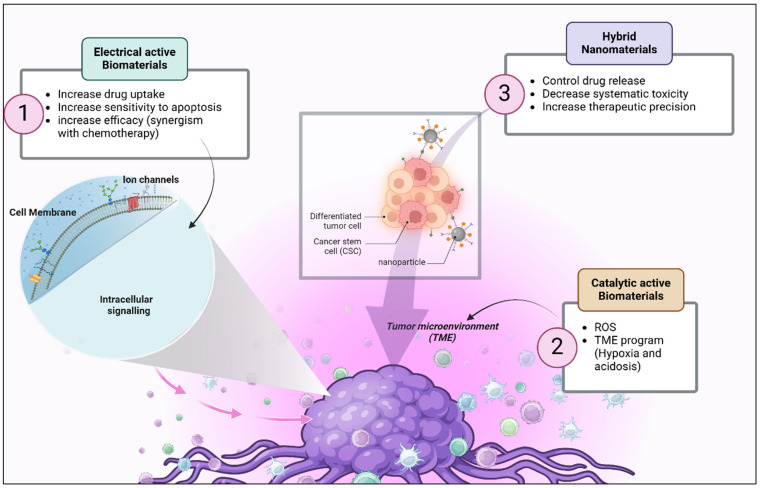
Different biomaterials targeting the tumor microenvironment (TME) to enhance cancer therapy. Electrically active biomaterials (green) increase drug uptake, enhance apoptosis sensitivity, and synergize with chemotherapy by interacting with ion channels on the cell membrane, affecting intracellular signaling. Catalytically active biomaterials (brown) modulate the TME by generating reactive oxygen species (ROS) and influencing hypoxia and acidosis. Hybrid nanomaterials (purple) improve drug delivery by controlling drug release, reducing systemic toxicity, and increasing therapeutic precision. The central illustration represents the TME, showing a tumor mass containing cancer stem cells (CSCs) and differentiated tumor cells, with nanoparticles interacting to improve treatment outcomes. Created in BioRender. Babiker, R. (2025) https://BioRender.com/v5pdxl2, accessed on 27 April 2025.

Hence, multifunctional biomaterials, arising from the fusion of materials science and biomedicine, offer solutions to healthcare challenges. Their catalytic capabilities offer possibilities for new and advanced personalized therapies. With advancements in technology, these materials are increasingly being explored for their role in overcoming drug resistance in cancer (Figure 4, Table 2).

## 4. Biomaterials and Their Potential in Overcoming Drug Resistance

Overcoming drug resistance remains a major challenge in cancer treatment, as cancer cells enable the development of mechanisms to elude therapy. Biomaterials and electrostimulation-based techniques offer promising approaches to counteract this resistance. The application of electric fields and specific materials has proven effective for disrupting cancer survival pathways, enhancing drug transport efficiency, and modifying the tumor microenvironment. This section explores how electrical properties can help combat drug resistance by stimulating cancer cells and integrating bioelectric targeting with chemotherapy.

### 4.1. Electrostimulation and Cancer Cells

Cells can respond to currents or fields through electrostimulation, which can affect their behavior at the cellular level. Cancer cells have the characteristic of being especially sensitive to direct electrostimulation [79]. This approach has shown promise in tackling drug resistance by interrupting pathways that contribute to resistance. It also improves the uptake of chemotherapy drugs within the cells.

#### 4.1.1. Enhancing Drug Penetration into Cancer Cells by Disrupting the Cell Membrane

The use of fields can change the permeability and structure of cancer cell membranes through a process known as electroporation, in which pores are created in the cell membrane, allowing drugs to pass through them. Electroporation has been utilized in both preclinical settings to enhance the administration of small-molecule medications, as well as DNA and RNA treatments. Electrostimulation offers a benefit by tackling the resistance caused by efflux pumps in cancer cells, enhancing drug levels, and ultimately improving treatment effectiveness [80]. Research has demonstrated that pulsed electric fields can boost the uptake of doxorubicin in drug-resistant tumors, thereby increasing its cytotoxic impact [81].

#### 4.1.2. Sensitizing Cancer Cells to Therapy by Modulating Ion Channels and Membrane Potential

In cancer cells, ion channel activity and membrane potential differ from those of normal cells, playing a role in their survival, growth, and resistance development [82]. Ion channels, such as calcium and potassium channels, can be affected by electrostimulation, which can regulate their activity and impact the signaling pathways related to drug resistance [83]. For instance, enhanced sensitivity of cells to apoptosis through changes in calcium influx has been observed, while adjusting potassium channels may disrupt balance and growth.

Bioelectric targeting takes advantage of electrically active materials to affect cellular signaling pathways that are associated with drug resistance. These materials work by engaging cells at the bioelectric level, changing important processes that lead to resistance.

##### Regulation of Cellular Signaling Pathways Through Electrically Active Materials

Furthermore, cancer cells can be subjected to localized electrical stimulation using electrically active materials such as conductive polymers and nanomaterials. These materials interact with the bioelectric properties of cancer cells and interfere with their cellular signaling pathways involved in resistance. For example, conductive polymers such as polyaniline and PEDOT:PSS have been employed to transmit electrical signals that regulate apoptosis-related pathways [84], thereby rendering chemotherapy-resistant cancer cells sensitive again. Gold nanorods, which function as metallic nanostructures, can also be integrated into an electrostimulation platform to apply a localized electric field that disrupts cancer cell signaling [85].

Examples of Electrically Active Materials in Localized Therapy:

(a) Conductive hydrogels: Soft, biocompatible materials with electrical conductivity that can provide localized electrical stimulation to specific tumors. They can enhance the treatment of resistant tumors. For instance, conductive hydrogels incorporated with anticancer drugs can release both drugs and electrical signals, exerting a synergistic effect on resistant tumors [86].
(b) Nanowires: Silicon and carbon nanowires have been used to transfer electrical pulses to cancer cells, while conductive nanowires have been employed to control cellular bioelectric properties due to their nanoscale dimensions. Studies have shown that stimulation with nanowires increases mitochondrial dysfunction in resistant cancer cells and thereby enhances apoptosis [87].

### 4.2. Synergizing Electrostimulation with Chemotherapy

Numerous scientists have revealed that the combination of electrical stimulation and chemotherapy is very promising in overcoming drug resistance. The electrical properties of the cell can thus work in synergy with chemotherapeutic agents to enhance their efficacy by sensitizing cancer cells to treatment, disrupting the mechanisms of resistance, and improving the delivery of drugs [79]. There are several ways in which electrical stimulation can enhance the therapeutic impact of chemotherapy. First, it increases cell membrane permeability, ensuring that chemotherapeutic agents enter the cell. Second, it blocks the activity of efflux pumps, such as P-glycoprotein, which are frequently overexpressed in resistant cancer cells to reduce the number of chemotherapeutic agents that remain in the cell to exert their cytotoxic effect [88]. It has been found that low-frequency electrical fields, when combined with paclitaxel or cisplatin, are very efficient in killing cancer cells in drug-resistant tumors [89].

In addition to electrostimulation strategies, the use of chemical inhibitors targeting efflux pathways has emerged as a complementary approach. Compounds such as CM082, a multi-targeted tyrosine kinase inhibitor, have demonstrated potential in modulating the tumor microenvironment and inhibiting the efflux mechanisms of ABCG2 (ATP-binding cassette transporter G2), thereby sensitizing resistant tumors to chemotherapy. The combination of this chemical inhibitor with electrostimulation techniques could provide a synergistic effect in overcoming drug resistance [90].

The synergistic effects of electrical stimulation and chemotherapy have been shown in several studies. For instance, Tumor Treating Fields (TTFields), a technology that has received FDA approval to use low-intensity alternating electric fields, has been found to improve the survival of patients with glioblastoma when used alongside temozolomide [91]. In addition, the use of pulsed electric fields in electrochemotherapy has been found to increase the effectiveness of bleomycin and cisplatin in various tumor models [92].

Electrical stimulation can also disrupt the tumor microenvironment to increase the effectiveness of chemotherapy. For example, electrical fields help normalize tumor vasculature and disrupt stromal cell interactions, thereby improving drug delivery to the tumor core. Moreover, the bioelectric modulation of immune cells within the TME can enhance the anti-tumor immune response, contributing to overall therapeutic effects.

### 4.3. Future Directions and Challenges

The integration of electrical properties into cancer therapy is a new approach to overcoming drug resistance. However, there are several challenges, including the optimization of electrical stimulation parameters, assurance of the biocompatibility of electrically active materials, and translation of preclinical findings into clinical applications. These challenges are expected to be addressed by advances in nanotechnology and material science, which are expected to enable the development of more effective and personalized electrostimulation-based therapies.

The use of electrical properties, whether through direct stimulation or electrically active materials, provides a multifaceted approach to addressing drug resistance in cancer through cellular disruption, enhanced drug delivery, and chemotherapy synergy. These strategies have the potential to revolutionize cancer treatment and enhance outcomes for patients with resistant tumors. Additional research and clinical trials are required to achieve the full potential of this innovative oncology approach.

## 5. Catalytic Biomaterials and Their Potential in Overcoming Drug Resistance

Another strategy to counter resistance involves the catalytic properties of biomaterials. Catalytic nanomaterials can modify the tumor microenvironment (TME), generate reactive oxygen species (ROS), enhance drug activation, and improve drug delivery [93]. This section will explore how catalytic properties can be leveraged to overcome drug resistance, focusing on catalytic nanomaterials, their interaction with the tumor microenvironment, and the application of specific catalysts.

Catalytic nanomaterials work by exploiting their catalytic activity to counteract cancer cell resistance mechanisms. These materials, often engineered at the nanoscale, are designed to interact with biological systems, producing biochemical effects that interfere with cancer cell survival and resistance. Furthermore, catalytic nanomaterials can modify the TME to counteract these resistance factors [94].

### 5.1. Catalytic Biomaterial and ROS

One of the most promising strategies for overcoming drug resistance using catalytic nanomaterials is the generation of reactive oxygen species (ROS) [95]. Therapy-resistant cancer cells often exhibit heightened antioxidant defense mechanisms, such as increased levels of glutathione (GSH) and catalase. Catalytic nanomaterials, such as cerium oxide nanoparticles, deplete these antioxidants and enhance ROS production, exploiting this vulnerability [96]. This targeted oxidative stress selectively kills resistant cancer cells while minimizing the risk to normal tissues. Iron oxide nanoparticles (IONPs) and manganese dioxide nanoparticles have been studied for their ability to generate ROS in response to tumor-related stimuli, such as low pH or elevated hydrogen peroxide levels in the TME [97]. These properties make them excellent candidates for enhancing cancer therapy efficacy by inducing oxidative stress in resistant cancer cells.

### 5.2. Catalytic Enhancement of Drug Activation In Situ

Catalytic nanomaterials can also improve prodrug activation within the tumor microenvironment. A prodrug is typically an inactive or weakly active drug that becomes active through metabolic or chemical processes. For example, platinum-based nanoparticles can enhance the conversion of cisplatin prodrugs into their active state at the tumor site, reducing systemic toxicity [98]. Enzyme-mimetic nanomaterials, such as glucose oxidase-conjugated gold nanoparticles, can catalyze hydrogen peroxide production from glucose. The resulting hydrogen peroxide is toxic to cancer cells, thereby increasing drug efficacy [99].

### 5.3. Tumor Microenvironment Reprogramming

The tumor microenvironment (TME) is a crucial factor in drug resistance, as it provides cancer cells with an environment that allows them to survive and resist therapy. Catalytic nanomaterials can modify the TME to counteract these resistance factors.

#### 5.3.1. Catalysis-Driven Normalization of the Acidic or Hypoxic Microenvironment

Key factors contributing to drug resistance include hypoxia and acidity within the TME. Tumor cells rely on anaerobic metabolism, leading to excessive lactic acid production and an acidic environment. This acidity can inactivate pH-dependent drugs and promote resistance.

Catalytic nanomaterials, such as manganese dioxide nanoparticles, help counteract this issue by buffering acidity and generating oxygen through catalytic reactions [100]. By restoring pH and oxygen levels, these materials improve the efficacy of oxygen-dependent treatments like radiation therapy and certain chemotherapies. For example, MOFs with catalase-like activity have been modified to degrade hydrogen peroxide into water and oxygen for the treatment of hypoxia [101]. This ‘normalization’ of the TME also improves drug efficacy while simultaneously reducing the invasiveness of cancer cells.

#### 5.3.2. Enzymatic Activity to Degrade Extracellular Matrix and Improve Drug Penetration

The dense extracellular matrix (ECM) in tumors presents a barrier to drug delivery, limiting therapeutic agent penetration into the tumor core. Catalytic nanomaterials with enzymatic activity, such as collagenase-mimetic nanoparticles, can degrade ECM components like collagen and hyaluronic acid [102]. This degradation enhances tissue permeability, improving drug accessibility and therapeutic outcomes.

Enzymatic nanoparticles mimicking matrix metalloproteinases (MMPs) have also been developed to degrade the ECM, breaking down the tumor’s structural integrity and making cancer cells more accessible to chemotherapeutic agents [103].

### 5.4. Case Studies

#### 5.4.1. Catalysts Based on Platinum in Combination Therapies

Cisplatin and carboplatin are widely used chemotherapeutic drugs but often lose effectiveness due to drug resistance. Platinum-based catalysts are being developed to enhance drug delivery and generate ROS, increasing cytotoxicity against resistant cancer cells.

For example, platinum nanoclusters grafted with tumor-homing ligands have demonstrated enhanced specificity and efficacy in treating resistant tumors in initial animal studies [104]. The combination of platinum catalysts with other ROS inducers, such as hydrogen peroxide, is believed to further enhance therapy by amplifying oxidative stress.

#### 5.4.2. Iron Oxide Nanoparticles as Ferroptosis Promoters

Ferroptosis is an iron-dependent form of cell death characterized by the accumulation of lipid-based peroxides [105]. Iron oxide nanoparticles (IONPs) promote ferroptosis by catalyzing the Fenton reaction, which uses hydrogen peroxide to generate highly reactive hydroxyl radicals. These radicals induce lipid peroxidation, ultimately triggering ferroptotic cell death in cancer cells [106].

The combination of IONPs with known ferroptosis inducers, such as erastin, has been shown to enhance the efficacy of both agents. Studies indicate that IONPs effectively combat resistance to chemotherapy and radiotherapy in various cancers, including glioblastoma and pancreatic cancer [107].

#### 5.4.3. Iron–Sulfur Cluster-Based Catalysts for Ferroptosis Induction

Iron–sulfur (Fe-S) clusters have been explored for their ability to amplify ferroptosis by upregulating the iron redox cycle.

For example, Fe-S nano-catalysts were developed, which induced ferroptosis more efficiently than traditional IONPs. These clusters increased lipid peroxidation while maintaining iron homeostasis, activating tumor cell death [108].

#### 5.4.4. Ruthenium and Platinum Hybrid Complexes for Evading Chemoresistance

Platinum-based drugs such as cisplatin often face resistance, limiting their effectiveness. To counter this, hybrid metal complexes encapsulating ruthenium and platinum have been developed to exploit their mechanisms of action.

A study reported that a ruthenium-platinum complex revealed increased DNA intercalation and ROS production as compared to cisplatin itself. This dual-metal approach enhanced apoptotic activity in lung and ovarian cancer models resistant to standard chemotherapy. The ruthenium improved cellular uptake and targeted mitochondria, while the platinum part retained its DNA-binding properties [109]. Clinical trials are underway to evaluate its efficacy in platinum-resistant tumors.

Catalytic nanomaterials are emerging agents that can improve the management of drug resistance and offer several advantages, including altering the TME, enhancing drug activation, and inducing oxidative stress. However, several challenges must be addressed, including optimizing the catalytic activity, ensuring biocompatibility, and minimizing side effects. This paper predicts that advancements in nanotechnology and materials science will overcome these challenges and facilitate the development of more effective catalytic therapies. When administered alongside other cancer treatments, catalytic nanomaterials represent a holistic approach to combating resistance. These materials target both the cellular and microenvironmental levels of resistance and may serve as a new standard of care for cancer treatment and patient response. Further research and clinical translation will be required to harness the full catalytic potential to overcome drug resistance.

## 6. Synergistic Role of Electrical and Catalytic Properties in Overcoming Drug Resistance

Cancer drug resistance thus calls for new and innovative strategies to improve the current therapeutic approaches. This paper presents a synergistic approach based on the combination of the electrical and catalytic properties of multifunctional biomaterials to treat cancer, in which the materials work simultaneously on various pathways to defeat cancer cell survival signals, enhance the delivery of drugs, and thus improve therapeutic outcomes.

### 6.1. Mechanisms of Electrical and Catalytic Activity

The use of a single material platform with both electrical and catalytic functionalities enables the material to address the problem of resistance in two ways. The electrostimulation and bioelectric modulation of the cellular membrane potential, ion channel activity, and intracellular signaling pathways comprise the electrical functionality of the system [110]. The catalytic functionality includes the generation of reactive oxygen species (ROS), alteration of the tumor microenvironment (TME), and activation of prodrugs. Thus, these mechanisms are effective in the fight against drug resistance.

An additional major benefit is the capacity to control the TME. Hypoxia, acidity, and the dense extracellular matrix (ECM) are the main factors that contribute to the development of drug resistance in the TME. Electrically active materials can enhance blood flow and oxygenation by applying electrical stimulation in a localized region, while catalytic materials can remedy pH and oxygen concentrations by participating in biochemical reactions [111]. This mechanism of action enhances the delivery of drugs and makes resistant tumors more sensitive to treatment.

The literature supports this phenomenon through the synthesis of nanohybrid materials, which are nanocomposites of electrically conducting components, such as carbon nanotubes, graphene, or conductive polymers, integrated with catalytic fillers including metallic nanoparticles or enzyme-mimicking nanomaterials [112]. These materials offer a single platform that can deliver the combined therapy to the target. For example, platinum nanoparticles have been loaded on the graphene oxide substrate to create a material with simultaneous high electrical conductivity and catalytic activity [113]. The graphene section enhances electrical interactions with cancer cells by altering ion channel function and membrane potential. However, the platinum nanoparticles generate ROS through a catalytic process, leading to oxidative stress and triggering apoptosis in resistant cancer cells [114]. It has been observed that these hybrid systems are very effective in improving the efficacy of chemotherapy and radiotherapy in resistant tumor models. In the same manner, gold nanorods incorporated into conductive hydrogels have been proposed to transport electric and catalytic treatment signals to the targeted cells [115]. These systems offer precise spatial and temporal control over treatment delivery, reducing systemic side effects and improving targeted drug administration.

### 6.2. Enhancing Drug Release

The electrical and catalytic characteristics of materials are very important in the delivery and release of anticancer drugs. These materials enable controlled and targeted drug delivery; the drugs that reach the tumor site arrive with the correct quantity of the drug in the system [116].

### 6.3. Improved Drug Delivery and Retention

Electrically active materials enhance the delivery of drugs by increasing cell membrane permeability through electroporation. This brief break in the cell membrane allows drugs to be internalized into the patient’s cells more effectively, without efflux pumps pumping them out [117]. Catalytic activity also enhances drug retention in the tissue by altering the tumor microenvironment, for example, by making the environment less acidic or by breaking down the extracellular matrix that prevents the penetration of drugs into the tumor [118].

**Figure 5:**
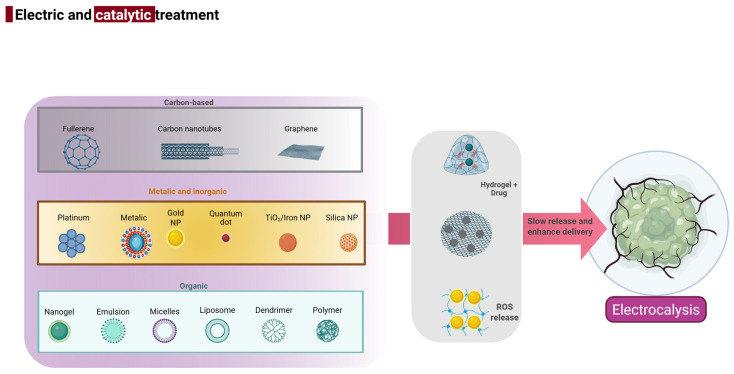
Various nanomaterial categories—carbon-based (e.g., fullerene, carbon nanotubes, and graphene), metallic and inorganic (e.g., platinum, gold nanoparticles, TiO_2_/iron oxide, and silica), and organic carriers (e.g., liposomes, dendrimers, and nanogels)—used for electro-catalytic cancer therapy. These platforms enable enhanced drug delivery through hydrogel encapsulation or reactive oxygen species (ROS) generation, facilitating slow release and improved targeting of tumor cells. The combined electric and catalytic approach supports sustained therapeutic action through electrocatalysis, leading to controlled tumor destruction.

The cooperative function of electrical and catalytic properties represents a novel approach to tackling the problem of drug resistance in cancer. These dual-functional materials act by targeting different mechanisms of resistance, influencing the tumor microenvironment, and enhancing drug delivery, and therefore, they hold promise for improving treatment outcomes (Figure 5). Further studies are needed to fully exploit their capabilities in precision oncology and translate them into clinical practice.

## 7. Challenges and Future Perspectives

Multifunctional biomaterials with integrated electrical and catalytic properties offer a promising strategy to combat drug resistance in cancer therapy. However, their clinical translation faces key challenges, including optimizing electrical stimulation parameters, ensuring biocompatibility, and bridging the gap between preclinical success and clinical application. Advances in nanotechnology and materials science are expected to drive the development of personalized, electrostimulation-based therapies that enhance drug delivery, disrupt resistant cancer cells, and improve chemotherapy outcomes. This section discusses the present problems, the future of multifunctional biomaterials, and their massive influence on cancer treatment.

### 7.1. Current Challenges

#### 7.1.1. Biocompatibility and Safety Issues

A major challenge in the fabrication of multifunctional biomaterials is ensuring that they are non-toxic and compatible with the body. Some electrically active and catalytic materials, such as metal nanoparticles and conductive polymers, may be toxic to cells, inducing inflammation or an immunogenic response when administered systemically. For instance, some metal oxides used in catalytic nanomaterials may leak toxic ions that are harmful to normal tissues [119]. Furthermore, ROS-generating materials, though very effective in inducing oxidative stress in cancer cells, can also affect the neighboring healthy cells if not well-targeted.

These problems can be solved through rigorous biocompatibility assessment and the use of surface treatments or coatings. These materials can have a biocompatible surface or be coated with biocompatible polymers or ligands to decrease their cytotoxicity while maintaining their therapeutic efficacy. Also, the use of materials with components that can be metabolized or absorbed will help in overcoming issues associated with long-term use.

#### 7.1.2. Scalability and Translation to Clinical Practice

The scalability of multifunctional biomaterials with good control over material properties remains a challenge. Many of these materials are sophisticated and require complex fabrication methods, such as nanoparticle synthesis or the integration of electrical and catalytic components, which are not easily scalable to commercial levels [120]. Furthermore, the reproducibility of these materials is of great importance for clinical applications because changes in the size, shape, and surface chemistry of these materials slightly alter their function and efficiency.

Translation to the clinic also presents regulatory challenges because multifunctional biomaterials are rather novel in their mechanism of action and interact with the biological environment to form a hybrid system. Thus, their effectiveness and safety must be proven through rigorous preclinical and clinical trials, a time- and resource-consuming process. The lack of standardization in the evaluation of these materials adds complexity to the situation.

### 7.2. Future Directions

#### 7.2.1. Integration of Immunotherapy and Personalized Medicine with Multifunctional Biomaterials

The application of multifunctional biomaterials in immunotherapy is another promising approach for future cancer treatments. These materials work with the immune system to help it recognize and attack tumors, and they enhance the effects of immunotherapies, such as anti-PD-1/PD-L1 or CAR-T cell therapies, by modulating the immune response [121] and engaging immune checkpoint pathways. For example, catalytic materials produce reactive oxygen species (ROS) as adjuvants to enhance the immune system’s ability to detect and destroy tumors.

Individualized medicine is another area where multifunctional biomaterials can be useful. The development of materials that are specific to the genetic and phenotypic characteristics of individual tumors will greatly enhance the delivery of therapy. Smart materials that can recognize tumor-related stimuli, such as pH, enzymes, or hypoxia, can carry drugs or other therapeutic agents to the target accurately [122]. This approach reduces side effects and improves the quality of life of the patient.

#### 7.2.2. Evolution of Intelligent Materials with Self-Regulating Characteristics

The next generation of multifunctional biomaterials is expected to include intelligent materials that can change their properties according to the tumor microenvironment or external stimuli, e.g., electrical or light stimuli. For instance, self-regulating hydrogels can release drugs at rates dependent on the pH or oxygen concentration in the tumor microenvironment to provide sustained and targeted drug delivery [123].

Additionally, the development of nanotechnology and artificial intelligence may enable the development of intelligent biomaterials that can sense their environment and provide feedback information. These materials may also sense alterations in tumor shapes, including the development of resistance, and modify their functions accordingly. Such changes are likely to shift cancer treatment toward highly dynamic and adaptive approaches.

#### 7.2.3. Tuning Nanomechanical and Rheological Properties for Improved Drug Delivery

Recent advances highlight the importance of adjusting the nanomechanical and rheological properties of drug delivery systems to match the physical characteristics of the tumor extracellular matrix (ECM). By designing nanoparticles and hydrogels that mimic the stiffness, elasticity, and viscosity of tumor tissues, drug penetration, retention, and therapeutic efficacy can be significantly improved [124,125]. Dense and fibrotic ECM structures in drug-resistant tumors pose major barriers to chemotherapy, but ECM-mimicking carriers can bypass these mechanical barriers and facilitate deeper drug delivery [126]. Moreover, tailoring nanoparticle softness and surface adhesion enhances cellular uptake and disrupts mechanical resistance pathways, thereby sensitizing tumors to therapy [127]. Such strategies represent a promising direction for overcoming drug resistance through the mechanical adaptation of multifunctional biomaterials.

#### 7.2.4. Potential Effects

The most outstanding feature of multifunctional biomaterials in cancer therapy is that they can attack the problem of drug resistance from many points simultaneously. These materials are electrical and catalytic in nature and combat multiple resistance mechanisms, making the approach more comprehensive for overcoming drug resistance.

##### Innovative Influence on Cancer Treatment

Multifunctional biomaterials have the capacity to enhance conventional treatments, including chemotherapy, radiotherapy, and immunotherapy. These materials can enhance the delivery of drugs, modify the tumor microenvironment, and kill only resistant cancer cells, thus solving most of the problems of the current treatment methods. For example, the electrical components of these materials can enhance drug internalization into the cell, whereas the catalytic components can assist in the reversal of hypoxic and acidic conditions that make cancer cells resistant [128].

##### Effect on Drug Resistance Management

The potential of multifunctional biomaterials to act on several processes that lead to resistance, such as efflux pumps, hypoxia, and mutation, makes them an important solution in the control of resistance. Also, their compatibility with other forms of treatment, like immunotherapy and gene editing, opens a new field of use for these materials. The only drawback is that these materials must be used in combination with other therapies to prevent the development of resistance and improve overall treatment results.

##### The Possibility of Global Healthcare Improvements

In addition to their clinical relevance, multifunctional biomaterials can improve the quality of cancer care and decrease the cost of treatment by improving response to treatments and reducing the number of treatments required. They are flexible and can be produced at scale, which makes it possible to extend the front of advanced cancer care to underserved populations and thus address the issue of equity in global healthcare.

#### 7.2.5. Clinical Translation Status and Future Trials

The clinical translation of biomaterials is still in an early stage. However, Tumor Treating Fields (TTFields) technology stands out, having successfully completed clinical trials and received FDA approval for glioblastoma and mesothelioma treatment [129].

Other technologies include electrically active materials (e.g., conductive hydrogels, and nanowires), and catalytic nanomaterials are still limited to preclinical investigations and early-phase clinical trials [130].

At present, no multifunctional biomaterial-based platforms integrating both electrical and catalytic features have received full clinical approval. The ongoing challenge is the rigorous demonstration of biocompatibility, long-term safety, and therapeutic efficacy through comprehensive clinical studies. Future efforts must focus on advancing these strategies through clinical pipelines to fully realize their potential in drug-resistant cancer therapy.

## 8. Conclusions

Multifunctional biomaterials with integrated electrostimulation, catalytic, or synergistic properties offer a revolutionary approach to overcoming drug resistance in cancer therapy. By targeting multiple resistance mechanisms—such as genetic mutations, efflux pumps, hypoxia, acidosis, and tumor microenvironmental factors—these materials enhance chemotherapy efficacy, drug delivery, and cellular response modulation. However, challenges such as biocompatibility, scalability, and clinical translation must be addressed. Future advancements should focus on optimizing material design for improved safety and efficacy, integration with immunotherapy and personalized medicine, and navigating regulatory barriers for clinical adoption. The development of self-adaptive biomaterials capable of responding to tumor conditions in real time, alongside AI-driven precision oncology platforms, will revolutionize cancer treatment. These innovations have the potential to not only refine existing therapies but also shape the future of personalized, targeted treatment strategies, ultimately improving patient outcomes worldwide.

## Figures and Tables

**Table 1:** List of different modes of drug resistance mechanisms and their characteristics.

Drug Resistance Mechanism	Pathway	Resistance from Drug	References
Drug efflux pump	Upregulation of ABC transporters: P-gp, BCRP, and MRP	Doxorubicin, axitinib, bisantrene, sunitinib malate	[36]
	Overexpression of P-gp via TRPC5-mediated Ca^2^^+^ influx activating NFATc3	Adriamycin, paclitaxel, temozolomide	[37]
Inhibition of cell death	Mutations occur in anti-apoptotic genes: caspases, BCL-2 family, BCL-XL, BAX MCL-1, TP53	Gemcitabine, rituximab	[38]
	Overexpression of ABCB1 gene	Carboplatin, Taxol, VP-16	[39]
DNA damage repair	NHEJ pathway	Chemotherapy	[40]
	Loss of APC	Doxorubicin	[41]
	Reduced ER protein and DNA repair mechanism; upregulation of the IL-6/STAT3 pathway	Palbociclib	[42]
	Upregulation of BRCA1, BRCA2, Rad51 gene	Radioresistance	[43]
Drug target modification	BCR-ABL signaling	STI-571 (Abl tyrosine kinase inhibitor)	[27]
	EGFR (T790M), HER2,	Tyrosine kinase inhibitors (TKIs), imatinib	[26]
	Mutation in β-tubulin	Paclitaxel	[44]
Epigenetics	Overexpression of ABCB1 levels	Adriamycin	[33]
	Overexpression of BMP4	Cisplatin	[45]
	Downregulation of GAS5 levels	Adriamycin	[33]
Tumor-promoting inflammation	Activation of STAT3	Tyrosine kinase inhibitors	[46]
	Elevated expression of P-gp	Cisplatin	[47]
	Elevated expression of TGF-β	Sorafenib	[48]
Immune Evasion	PD-L1, MDSCs, Tregs	Immunotherapy (e.g., checkpoint inhibitors)	[49]
Genome mutations	Chromosomal instability	Paclitaxel, carboplatin	[50]
	Aneuploidy	Cisplatin	[51]

**Table 2:** List of multifunctional biomaterials along with markers for combating drug resistance.

Biomaterial	Drug Resistance Marker	Role of Markers	Mechanism	References
PLA and PEG nanoparticles	Transporter proteins (P-gp, MRP-1, MRP-2)	Efflux of drug from cancer cells, reducing drug concentration inside the cell	Deliver anticancer drugs without the use of P-gp	[67]
Nanotubes	HIF-1α	Induce hypoxia and decrease drug influx and promotes resistance	siRNA against HIF-1α reduces its activity	[68]
Dendrimers/magnetic nanoparticles	Survivin	Inhibits apoptosis and enhances cancer cell survival	Antisense survivin-loaded nanoparticles silence survivin expression to promote apoptosis	[69]
Mesoporous silica nanoparticles	Bcl-2	Anti-apoptotic gene that prevents cell death	siRNA-loaded MSNs suppress Bcl-2, enhancing chemotherapy-induced apoptosis	[70]
Poly(D,L-lactide-co-glycolide)	p53 mutations	Loss of tumor suppressor function, leading to unchecked cell growth	Deliver wild-type p53 DNA for restoring tumor suppressor activity	[71]
Liposomes	Transferrin receptor	Overexpressed in MDR cancer cells, aiding drug resistance	Liposome-conjugated transferrin targets cancer cells for direct drug uptake	[72]
Poly(beta-amino ester)	Intracellular pH	Low pH reduces drug effectiveness and uptake	Enhanced drug delivery even in acidic conditions	[72]
PEO-modified poly(ε-caprolactone)	Ceramide levels	Reduced ceramide levels prevent apoptosis in cancer cells	Increase ceramide levels to induce cancer cell death	[73]
Functionalized quantum dots plus magnetic iron oxide nanoparticles	EGFR	Promotes tumor growth and drug resistance	Targeted systemic delivery of EGFR antibodies for cancer therapy	[74]
PAMAM dendrimers	Folate receptors	Overexpressed in breast, kidney, ovary, lung, and brain cancers	Use excessive folate receptors for targeted drug delivery	[75]
Herceptin-dextran iron oxide nanoparticles	HER2/neu receptors	Overexpression leads to resistance in breast cancer	High accumulation in tumors to reduce tumor volume	[76]
PLGA-PEG nanoparticles	PSMA	Causes prostate cancer progression	Internalized by PSMA-positive cells, leading to targeted therapy	[77]
PEG-poly(ε-caprolactone) nanoparticles	Lipoprotein receptor-related protein	Overexpressed in gliomas and brain cancers, aiding drug resistance	Enables drug penetration through the blood–brain barrier	[78]

## Data Availability

All the data are presented in the manuscript.

## Abbreviations

ABC transporter	ATP-binding cassette transporter
BAX	B-cell lymphoma protein 2 (Bcl-2)-associated X protein
BCL	B-cell lymphoma 2
BCRP	Breast cancer resistance protein
CMI	Chronic myeloid leukemia
ECM	Extracellular matrix
EGFR	Epidermal growth factor receptor
Fe-NP	Iron nanoparticles
GSH	Glutathione
IONPs	Iron oxide nanoparticles
MDR	Resistance to multiple drugs
MOFs	Metal–organic frameworks
MRPs	Multidrug resistance-associated proteins
NK cells	Natural killer cells
NSCLC	Non-small cell lung cancer
PEDOT:PSS	Poly(3,4-ethylenedioxythiophene):polystyrene sulfonate
ROS	Reactive oxygen species
TiO_2_	Titanium dioxide
TKIs	Tyrosine kinase inhibitors
TME	Tumor microenvironment
WHO	World Health Organization
